# Ioduretted Syrup of Sarsaparilla

**Published:** 1843-11

**Authors:** 


*Ioduretted Syrup of Sarsaparilla.*—Ricord has just given a formula in the "Gazette de Santé" *for ioduretted syrup of sarsaparilla*, which is composed of a drach of iodide of potassium to about twelve ounces of sarsaparilla syrup, and which, as he says, is highly suitable as a remedy for *tertiary* syphilitic symptoms, or after the patient has been the subject of a long mercurial course. A tablespoonful may be taken three times daily.—*London Lancet.*

